# Effects of Pulse Current on Endurance Exercise and Its Anti-Fatigue Properties in the Hepatic Tissue of Trained Rats

**DOI:** 10.1371/journal.pone.0075093

**Published:** 2013-10-08

**Authors:** Qi Chang, Xinfang Miao, Xiaowei Ju, Lvgang Zhu, Changlin Huang, Tao Huang, Xincheng Zuo, Chunfang Gao

**Affiliations:** 1 Institute of Military Training Related Medical Science, The 150th Hospital of PLA, Luoyang, Henan, China; 2 Department of Internal Medicine, The General Logistics Department of PLA, Beijing, China; 3 Institute of Anus and Intestine, The 150th Hospital of PLA, Luoyang, Henan, China; Rutgers University, United States of America

## Abstract

Fatigue is synonymous with a wide spectrum of familiar physiological conditions, from pathology and general health, to sport and physical exercise. Strenuous, prolonged exercise training causes fatigue. Although several studies have investigated the effects of electrical stimulation frequency on muscle fatigue, the effects of percutaneous pulse current stimulation on fatigue in the hepatic tissue of trained rats is still unclear. In order to find an effective strategy to prevent fatigue or enhance recovery, the effects of pulse current on endurance exercise and its anti-fatigue properties in exercised rats were studied. Rats were subjected to one, three or five weeks of swimming exercise training. After exercise training, rats in the treated group received daily applications of pulse current. All rats were sacrificed after one, three or five weeks of swimming exercise, and the major biochemical indexes were measured in serum and liver. The results demonstrate that pulse current could prolong the exhaustion swimming time, as well as decrease serum ALT, AST and LD levels and liver MDA content. It also elevated serum LDH activity, liver SOD activity and glycogen content. Furthermore, pulse current increased the expression of Bcl-2 and decreased the expression of Bax. Taken together, these results show that pulse current can elevate endurance capacity and facilitate recovery from fatigue.

## Introduction

Regular physical exercise has many health benefits, including a reduced threat of all-cause mortality along with a reduced risk of cardiovascular disease, cancer, and diabetes [1], [2]. However, it is also clear that prolonged and intense exercise can result in fatigue. Physical fatigue is also called peripheral fatigue, and may be accompanied by a deterioration in performance [3]. Although a common phenomenon, the mechanisms involved in its development remain elusive. Numerous mechanisms and contributory factors have been implicated in the development of fatigue over the years, and include, amongst others, exercise itself as it promotes the consumption and depletion of energy sources such as glycogen. Exercise also causes the production and accumulation of metabolic products such as lactic acid and ammonia in the body. Intense exercise can produce an imbalance between the body’s oxidation and anti-oxidation systems [4]. Recovery from exercise-induced fatigue requires repairing the damage that has occurred in the body and prompting the elimination of the metabolic products that accumulated during exercise. Long-term accumulated fatigue can lead to chronic fatigue syndrome. As characterized by the most widely used case definition, chronic fatigue syndrome is disabling fatigue for six or more months with four out of eight core symptoms including: impaired memory or concentration, headaches, sore throat, lymph node pain, muscle pain, joint pain, unrefreshing sleep, and post-exertional malaise [5].

People have tried various strategies to improve the body’s strength and enhance recovery from fatigue, including the use of stimulants, which cause long-term harm to the body [6]. Electrical stimulation is also used to apply high-intensity training exercises on stimulated muscles while not being limited by cardiovascular stress, which is the case in voluntary training exercises. Several studies have investigated the effects of electrical stimulation frequency on muscle fatigue. Gondin et al. reported that the correlation between decline in muscle force and decline in muscle excitability capacity was dependent on electrical stimulation frequency [7]. Kesar et al. reported that electrical stimulation could increase muscle force output and counteract the effects of muscle fatigue [8].

Liver is one of the most important organs affected by prolonged exercise. Some studies reported that acute exercise induces an increase in MDA and a decrease in glutamine synthetase in liver homogenate [9]; long-term endurance exercise increases oxidative and mitochondrial stress in the liver [10]. However, the effects of percutaneous pulse current stimulation on anti-fatigue in the hepatic tissue of trained rats is still unclear.

Therefore, the primary purpose of the present study was to investigate the anti-fatigue effects of percutaneous pulse current stimulation on the hepatic tissue of exercise-induced fatigue models. Our findings show that, after exercise training, pulse current could elevate the endurance capacity and facilitate recovery from fatigue.

## Materials and Methods

### Animals

One hundred and fifty male Wistar rats (initial body weight, 203±8.35 g; 8 weeks old) were obtained from the laboratory animal center of Henan province. They were housed in an animal room at 22±2°C and 60±2% relative humidity and had free access to laboratory chow and tap water. After one week of pre-feeding, the animals were randomly divided into the control group (n = 24), the exercise training group (n = 24), the stimulation A group (n = 24), the stimulation B group (n = 24) and the stimulation C group (n = 24). All of the groups were given swimming training to establish an exercise-induced fatigue model except for the control group, then pulse current of 500 Hz, 1024 Hz and 2048 Hz were given to the stimulation A, B, and C groups, respectively.

### Construction of the Exercise-induced Fatigue Model

The swimming exercise performance test was used as previously described with some modifications [11]. A rat was taken out from each treatment group for swimming exercise and loaded to a constant weight corresponding to 5% of the individual body weight. The rats were individually placed in a columnar swimming pool (65 cm tall and radius 20 cm) with 40 cm deep water maintained at 27±1°C for 1 h/day, 6 days/week, for 5 weeks. After exercise training, rats in the treated groups received daily applications of a pulse current for 20 min over a period of 35 days. In the stimulation groups, frequencies of 500 Hz, 1024 Hz and 2048 Hz were used at an intensity of 10 mA. The endurance for each rat was measured as the swimming times recorded from the beginning to exhaustion, which was determined by observing a loss of coordinated movements and failure to return to the surface within 7 s. In the swimming period, the time spent floating, struggling and making necessary movements was also considered until exhaustion and possible drowning. Animal experiments conformed to the guidelines issued by the Institute of Military Training Related Medical Science of PLA for Laboratory Animals. The present study was performed with approval from by the Animal Ethics Committee of the Institute of Military Training Related Medical Science of PLA (Certification NO:0132). All surgery was performed under sodium pentobarbital anesthesia (Sigma, St. Louis, MO), and all efforts were made to minimize suffering.

### Histological Examination of Liver Tissue

Liver tissues were removed from the control and experimental groups at the end of experiment, and fixed in 10% buffered formalin and then embedded in paraffin. Paraffin-embedded samples were sectioned and underwent hematoxylin and eosin (H&E) staining as previously described [12].

### Immunohistochemistry

The livers were frozen and then cut into 10 µm sections. The sections were air-dried, fixed in ice-cold methanol for 10 min, rinsed in phosphate-buffered saline (PBS) and blocked with normal goat serum in incubation buffer (0.2% Tween20 and 0.1% bovine serum albumin in 0.15 M PBS). Incubation with primary antibodies (anti-Bax antibody, anti-Bcl-2 antibody) (Invitrogen, Carlsbad, CA) was performed in humidified chamber overnight at 4°C. Sections were subsequently washed in washing buffer (0.5% Tween20 in 0.15 M PBS) and incubated with a biotinylated conjugated secondary antibody (Invitrogen, Carlsbad, CA), followed by incubation with an avidin-biotin detection system (Vectastain ABC system). A diaminobenzidine (DAB) stain kit (Beyotime, Nantong, China) was used to detect a positive reaction by producing a brown color.

### Transmission Electron Microscope (TEM) Study

Liver samples treated with magnetic-core or magnetic-fluorescence nanoparticles were fixed with a 4% paraformaldehyde solution and dehydrated on graded concentrations of alcohol. Negatively stained specimens were placed on polyvinyl formal resin carbon-coated grids, and examined under Karl Zeiss EVO LS10 STEM electron microscope (Peabody, MA, USA).

### Sample Collection

Rats were deeply anesthetized with 10% chloral hydrate (3.5 mL/kg body weight) through intraperitoneal injection. Blood samples were collected in heparinized tubes from the femoral artery. Serum was centrifuged at 3,000 rpm for 10 min and stored at −80°C until use. And liver was quickly dissected and wash in ice-cold saline solution, then frozen in liquid nitrogen.

### Measuring Biochemical Parameters Related to Fatigue

Lactic acid (LA), lactate dehydrogenase (LDH), alanine aminotransferase (ALT), and aspartate aminotransferase (AST) were tested on a UV9100 spectrophotometer (Beijing Ruili Analytical Instrument Corp., Beijing, China) using commercial kits purchased from Nanjing Jiancheng Bioengineering Institute (Nanjing, China). And, determination of malondialdehyde (MDA) content, superoxide dismutase (SOD) activity, and glycogen content were performed according to the recommended procedures provided by the kits.

### Statistical Analysis

All values are expressed as the mean±SD. A paired Student’s t-test was used to assess the significance of differences between two mean values. *P*<0.05 was considered to be statistically significant.

## Results

### Effect of Pulse Current on Exhaustion Swimming Time in Rats

The mean swimming time to exhaustion is an important parameter of fatigue. Therefore, we investigated the effect of pulse current on the maximum swimming time of rats. As shown in Table 1, after one week exercise training, there were no significant differences among these five groups (*P*>0.05). However, after three weeks of exercise training, in the exercise training group, the mean swimming time to exhaustion was 100.08±11.61 min, while that in the stimulation B group was 140.96±12.56 min to exhaustion, i.e. 40.81% longer than that in the exercise training group (*P*<0.05). Additionally, after five weeks of exercise training, the mean swimming time to exhaustion in the stimulation B group was also markedly prolonged as compared with the exercise training group (*P*<0.05).

**Table 1:** Effect of pulse current on swimming time to exhaustion of rats.

groups	Swimming time to exhaustion (min)		
	1 W	3 W	5 W
control	154.42±13.43	155.76±13.40	156.45±12.44
training	142.05±16.54(1)	100.08±11.61(3)	81.72±13.27(3)
A	145.80±13.20(1) (2)	123.05±16.47(3) (4)	102.55±15.74(3) (4)
B	150.46±13.88(1) (2)	140.96±12.56(3) (4) (5)	122.58±18.14(3) (4) (5)
C	149.53±12.04(1) (2)	122.55±17.02(3) (4) (6)	103.11±16.20(3) (4) (6)

(1) Compared with control group: P>0.05;

(2) Compared with training group, P>0.05;

(3) Compared with control group: P<0.05;

(4) Compared with training group, P<0.05;

(5) Compared with A and C group: P<0.05;

(6) Compared with A group: P>0.05.

### Effects of Pulse Current on ALT, AST, LD, and LDA in Blood Serum

Blood samples were collected soon after the last forced swimming test to analyze the serum components. As shown in Table 2, after exercise training, rats in the exercise training group showed a significant increase in ALT, AST, LD, and LDH levels, as compared to the control group and stimulation groups (*P*<0.05). Meanwhile, the levels of ALT, AST, and LD in the stimulation B group were significantly decreased, as compared with the stimulation A and C groups. There was no significant difference observed in ALT, AST, and LD levels between the stimulation A and C groups.

**Table 2:** Effect of pulse current on ALT, AST, LD and LDA in blood serum.

Index	Group	week		
		1 W	3 W	5 W
ALT(U/L)	control	38.62±3.11	38.75±2.92	38.88±2.70
	training	58.25±5.82(1) (2)	65.38±5.12(1) (2)	73.00±6.37(1) (2)
	A	47.86±6.38(1) (3)	55.88±3.91(1) (3)	66.75±5.28(1) (3)
	B	39.50±5.04(5)	46.00±5.34(1)	58.62±7.00(1)
	C	51.75±5.26(1) (3) (4)	59.62±5.42(1) (3) (4)	66.25±4.68(1) (3) (4)
AST(U/L)	control	107.25±13.02	108.88±16.06	106.50±13.79
	training	149.88±14.32(1) (2)	169.25±14.60(1) (2)	187.25±16.10(1) (2)
	A	129.75±13.49(1) (3)	150.25±17.13(1) (3)	172.00±14.01(1) (3)
	B	110.12±9.85	128.00±19.95(1)	153.50±11.56(1)
	C	127.88±15.60(1) (3) (4)	148.12±15.68(1) (3) (4)	170.12±12.48(1) (3) (4)
LD(mmol/L)	control	6.52±1.99	6.38±1.99	6.33±1.94
	training	14.75±2.56(1) (2)	18.89±2.50(1) (2)	22.18±2.14(1) (2)
	A	11.13±1.82(1) (3)	16.17±2.58(1) (3)	19.23±2.50(1) (3)
	B	8.05±1.80(5)	12.69±1.95(1)	16.04±1.97(1)
	C	10.91±2.06(1) (3) (4)	15.95±2.13(1) (3) (4)	18.68±2.42(1) (3) (4)
LDH (U/L)	control	1059.75±103.88	1056.88±95.72	1058.38±99.31
	training	1335.50±126.81(1) (2)	1476.50±105.30(1) (2)	1831.00±106.06(1) (2)
	A	1108.12±81.54(5) (6)	1261.38±144.72(1) (6)	1523.00±125.50(1)
	B	1072.25±93.24(5) (6)	1185.38±125.12(1) (6)	1421.63±111.77(1) (6)
	C	1097.25±77.80(5) (6)	1232.75±137.54(1) (6)	1511.75±109.66(1) (6)

(1) Compared with control group: P<0.05;

(2) Compared with stimulating groups: P<0.05;

(3) Compared with stimulating B group: P<0.05;

(4) Compared with stimulating A group: P>0.05;

(5) Compared with control group: P>0.05;

(6) Comparison between stimulating groups: P>0.05.

### Effects of Pulse Current on SOD, MDA, and Glycogen Contents of the Liver

Based on the above results, a suitable stimulating frequency (1024 Hz) of pulse current was determined. Then, we investigated the effects of pulse current on the SOD, MDA, and glycogen contents of hepatic tissue. The results are shown in Table 3. After three weeks and five weeks of exercise training, compared to the control group, the SOD level of liver homogenates was clearly reduced in the exercise training group (*P*<0.05). Also, the stimulation B group showed a significant increase in SOD activity in liver homogenates, as compared with the exercise training group (*P*<0.05). The MDA contents of liver homogenates in the stimulation B group were higher than in the control group (*P*<0.05) and lower than in the exercise training group (*P*<0.05). The glycogen levels in stimulation group were significantly lower than in the control group and were much higher than in the exercise training group (*P*<0.05); glycogen followed a trend similar to SOD levels.

**Table 3:** Effect of pulse current on SOD, MDA and glycogen contents of liver.

Index	Group	week		
		1 W	3 W	5 W
SOD(U/mgprot)	control	323.96±23.72	322.22±21.24	314.88±19.33
	Training	259.90±24.87(2)	226.70±17.65(2)	211.40±21.74(2)
	B	322.94±20.90(1) (3)	290.79±15.02(2) (3)	262.44±23.00(2) (3)
MDA(U/mgprot)	control	7.19±0.45	7.24±0.53	7.15±0.47
	Training	9.60±0.70(2)	11.18±0.63(2)	12.37±0.67(2)
	B	7.25±0.52(1) (3)	8.12±0.57(2) (3)	9.59±0.81(2) (3)
Glycogen(mg/g)	control	20.80±1.34	20.82±1.07	20.89±1.61
	Training	15.67±1.80(2)	12.37±1.56(2)	10.63±1.47(2)
	B	21.27±1.80(1) (3)	18.56±1.53(2) (3)	16.18±1.54(2) (3)

(1) Compared with control group: P>0.05;

(2) Compared with control group: P<0.05;

(3) Compared with exercise training group: P<0.05.

### Effects of Pulse Current on the Ultrastructure of Liver Tissue

**Figure 1:**
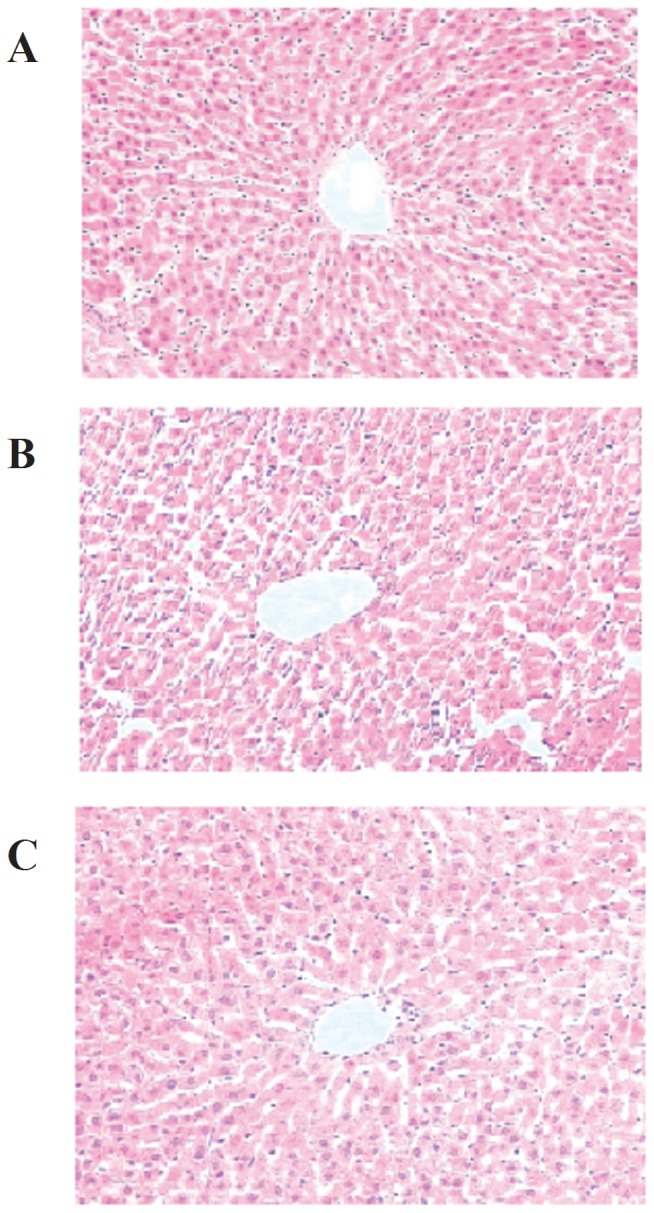
Sections of liver tissue were stained with hematoxylin and eosin. A, control group; B, five weeks exercise-training group; C, five weeks stimulating group.

**Figure 2:**
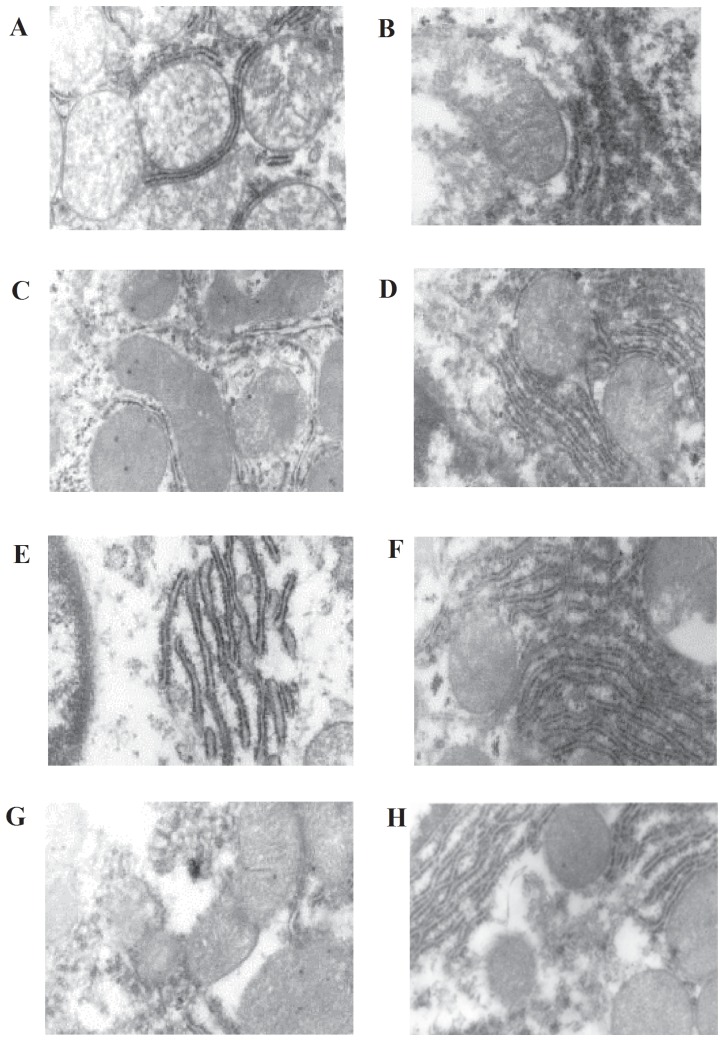
TEM of liver tissue. A and B, control group; C, one week exercise-training group; D, one week stimulating group; E, three weeks exercise-training group; F, three weeks stimulating group; G, five weeks exercise-training group; H, five weeks stimulating group.

Hepatic tissue histopathology showed that along with a deepening degree of exercise-induced fatigue, the pathological changes were gradually aggravated. The profile of some hepatic lobules was changed, liver cells demonstrated significantly swell, liver sinusoidal was narrow or even disappear, the nuclear was small and the vessels were expanded between interlobules and portal area (Figure 1B). While, after five weeks stimulating, liver cells were mildly swell, there was no significant void degeneration, and the vessels were slightly expanded between interlobules and portal area (Figure 1C). In addition, the ultrastructure of liver tissues was obtained by TEM. Compared with control group (Figure 2A and 2B), after one week exercise-training and stimulating, the structure of liver cells was integrity, mitochondria and endoplasmic reticulum were abundant, mitochondria crista had no obvious changes and the glycogen was slightly reduced (Figure 2C and 2D). After three weeks exercise-training, liver cells and mitochondria were swell, hepatic sinusoid was narrow, and rough endoplasmic reticulum (RER) was expanded (Figure 2E); while, after three weeks stimulating, liver cells and mitochondria were slightly swell, RER was mildly expanded (Figure 2F). Then after five weeks exercise-training, the mitochondria displayed heteromorphosis; mitochondrial membrane structure was broken, RER and glycogen were significantly decreased (Figure 2G). The mitochondria and RER of rat hepatocytes in five weeks stimulation group also showed damage (Figure 2H), but the degree of damage was milder than in five weeks exercise-training group.

### Pulse Current Increases the Expression of Bcl-2 and Decreases the Expression of Bax

**Figure 3:**
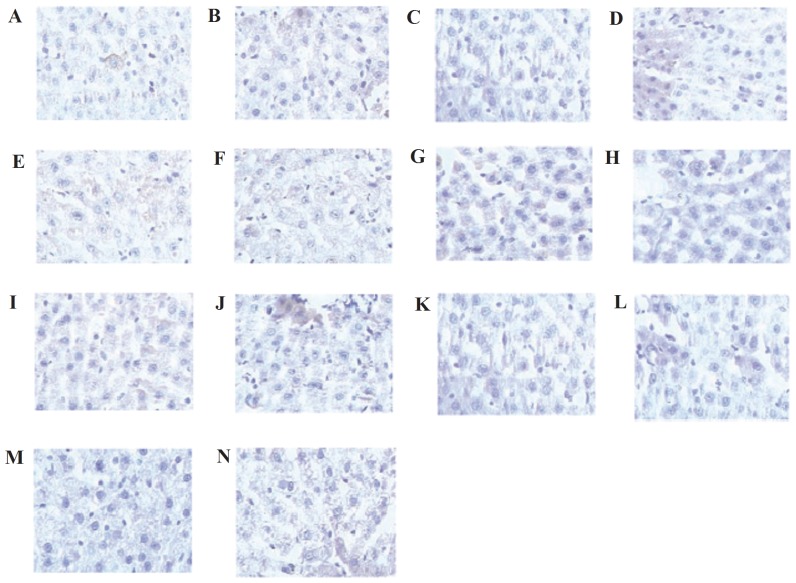
Immunohistochemical staining of Bcl-2 and Bax in liver tissue. A, Bcl-2 of control group; B, Bax of control group; C, Bcl-2 of one week exercise-training group; D, Bax of one week exercise-training group; E, Bcl-2 of one week stimulating group; F, Bax of one week stimulating group; G, Bcl-2 of three weeks exercise-training group; H, Bax of three weeks exercise-training group; I, Bcl-2 of three weeks stimulating group; J, Bax of three weeks stimulating group; K, Bcl-2 of five weeks exercise-training group; L, Bax of five weeks exercise-training group; M, Bcl-2 of five weeks stimulating group; N, Bax of five weeks stimulating group.

We found that pulse current could attenuate liver damage. To further study the anti-injury mechanism of pulse current, we investigated the Bcl-2 and Bax of liver tissue by immunohistochemistry. As shown in Figure 3, Bcl-2 and Bax were detected in different groups. Then quantitative analysis of Bcl-2 and Bax were performed, the results were shown in Table 4, after one week of exercise training, the expression of Bcl-2 and Bax in each group was not different (*P*>0.05). However, after five weeks of exercise training, the expression of Bcl-2 in the stimulation B group was significantly higher than that in the exercise training group (*P*<0.05) and was obviously lower than that in the control group (*P*<0.05). On the contrary, the Bax expression levels in the stimulation B group were higher than those in the control group (*P*<0.05) and lower than those in the exercise training group (*P*<0.05). During the period of training, the Bcl-2/Bax ratio in the exercise training group was lower than in the control group and the stimulation group (*P*<0.05). These results suggest that pulse current may increase the expression of Bcl-2 and decrease the expression of Bax.

**Table 4:** Effect of pulse current on the expression of Bcl-2 and Bax.

week	Group	Bcl-2 (IOD)	Bax (IOD)	Bcl-2/Bax
1 W	control	63.40±6.73	68.42±5.84	0.9250±0.0316
	training	61.21±5.65(1) (2)	89.95±6.41(3)	0.6788±0.0295(3)
	B	62.89±5.04(1) (2)	68.03±6.06(1) (4)	0.6788±0.0295(3)
3 W	control	65.38±3.96	69.86±5.03	0.9349±0.0333
	training	54.39±5.68(3)	107.99±4.69(3)	0.5025±0.0413(3)
	B	61.60±5.79(1) (4)	92.63±9.53(3) (4)	0.6675±0.0570(3) (4)
5 W	control	62.71±4.26	67.86±4.53	0.9238±0.0350
	training	48.10±5.05(3)	120.08±4.08(3)	0.4000±0.0457(3)
	B	54.43±4.84(3) (4)	109.64±7.03(3) (4)	0.4963±0.0385(3) (4)

(1) Compared with control group: P>0.05;

(2) Compared with training group: P>0.05;

(3) Compared with control group: P<0.05;

(4) Compared with training group: P<0.05.

## Discussion

In this study, we constructed a rat swimming model to investigate the effects of pulse current on exercise-induced fatigue. The results demonstrate that pulse current prolonged the exhaustion swimming time, decreased serum ALT, AST, and LD levels and reduced the liver MDA content. It also elevated serum LDH activity, liver SOD activity and glycogen content. Furthermore, pulse current increased the expression of Bcl-2 and decreased the expression of Bax. Taken together, pulse current was found to increase endurance capacity and facilitate recovery from fatigue.

Among the variety of physical exercises most used in studies involving animals, treadmill running and swimming stand out [13]. Although there are still doubts regarding which exercise is the most suitable to avoid unnecessary stress to the animals, swimming to exhaustion is an experimental exercise model which works well for evaluating the endurance capacity of rats and gives high reproducibility [14]. Reduced susceptibility to fatigue is correlated with longer swimming times. In this study, we constructed a swimming-induced fatigue model in rats to investigate the effects of pulse current on endurance capacity and its anti-fatigue properties in trained rats. Our study found that pulse current could prolong the swimming time of rats, suggesting that pulse current provides an anti-fatigue effect.

ALT and AST measurements are important for the assessment of liver damage [15]. Our study found that pulse current could decrease the levels of serum ALT and AST, which suggests that pulse current prevents fatigue by decreasing the metabolic rate of ALT and AST in serum. The level of blood lactate is dependent on the rate of lactate production by glycolysis and its utilization as a substrate, and lactate production increases with the degree of exercise intensity [16]. Therefore, blood lactate is considered an index of exercise intensity. Higher LA concentrations were observed in the exercise training group and lower LA concentrations were found in the stimulation groups, which presumably reflects a lower intramuscular lactate concentration and increased relative contribution of aerobic metabolism to ATP production during an exercise session [17].

LDH is known to be an accurate indicator of muscle damage as it catalyzes the inter-conversion of pyruvate and lactate [18]. Our results show that rats in the stimulation groups had a higher degree in LDH activity after exercise than those in the control group. This suggests that pulse current prevents fatigue by accelerating the metabolic rate of lactic acid in muscle.

SOD is regarded as one of the first lines of defense of the anti-oxidant enzyme system against reactive oxygen species generated during exhaustive physical exercise [19]. Previous studies have reported that SOD activity in serum is increased with oxidative stress due to exercise-induced fatigue [20]. In our study, SOD activity after pulse current stimulation was significantly higher compared to the exercise training group. This result indicates that pulse current might have beneficial effects in attenuating the oxidative stress caused by exhaustive physical exercise.

MDA is one of the most sensitive biomarkers of oxidative stress. It is the main product of lipid peroxidation induced by free radicals and can be found under physiological conditions in low concentrations. Most studies have shown that endurance exercise causes an increase in MDA [21], [22]. In this study, we found that pulse current could decrease the content of MDA, which indicates that pulse current can reduce the lipid peroxidation damage caused by free radicals, and thereby protect the integrity of the liver cell membrane. This maintains normal physiology of the liver, enhances athletic ability, and promotes the recovery process after exercise in mice [23].

Energy storage and supply is another important factor related to exercise performance. In terms of energy expenditure with exercise, rapid ATP consumption and energy deficiency is a critical cause of physical fatigue. Glycogen is the predominant source of glycolysis for ATP production. Therefore, glycogen storage directly affects exercise ability [24]. Our results show that pulse current elevated the liver glycogen content. One study has reported that at an intermediate exercise intensity, exhaustion is due mostly to the depletion of muscle glycogen, and repletion of liver glycogen may be the rate-limiting factor in the restoration of the capacity for prolonged strenuous exercise [25]. These results show that pulse current can enhance endurance by increasing the storage of liver glycogen.

Bax is a pro-apoptotic member of the Bcl-2 family that interferes with mitochondrial function by forming pores in the outer mitochondria membrane [26]. Bax expression results in cytochrome *c* release, finally leading to the cleavage of proteins essential for cell survival. However, this process is tightly controlled antagonistically by anti-apoptotic members of the Bcl-2 protein family, which can inhibit Bax activation through a direct interaction [27]. In this study, we found that pulse current could upregulate the expression of Bcl-2 and downregulate the expression of Bax, indicating that pulse current can facilitate recovery from fatigue by regulating the expression of Bcl-2 and Bax.

In conclusion, this report demonstrates that pulse current can enhance exercise endurance capacity and facilitate recovery from fatigue. Therefore, it may provide a new, effective, and powerful strategy to treat exercise-induced fatigue.
